# Protected Research Information Management Environment (PRIME) provides a secure open source data management option for clinical and scientific research

**DOI:** 10.1186/1471-2105-12-S7-A8

**Published:** 2011-08-05

**Authors:** Teeradache Viangteeravat, Venkateswara Ra Nagisetty, Matthew N Anyanwu, Emin Kuscu, Ian M Brooks, Chanchai S McDonald

**Affiliations:** 1 Biomedical Information Sciences Unit, Clinical & Translational Science Institute, University of Tennessee Health Science Center, Memphis, TN 38163

## Meeting abstract

The Biomedical Information Sciences Unit (BISU) of the UT-CTSI developed a secure, centralized information management system that is accessed through web applications. This system, called Slim-Prim [1,2], is mounted on an Oracle server housed within the UT-ITS and currently hosts three multi-site clinical trials and several institutional trials. In addition, Slim-Prim houses databases of clinical and laboratory information and provides a centralized location for several physician-generated surveys. In the majority of these cases users have approached the BISU and asked for assistance with their data management needs. Slim-Prim [1,2] and the BISU provide a cost effective mechanism for funded groups to handle their data management needs. However, we are also aware that many smaller groups are in desperate need of secure yet flexible database solutions; thus there is the need for a portable, secure and open source instance of Slim-Prim. To this end we have developed and released an open source instance of the Slim-Prim system under the Affero GPL v3 (http://www.gnu.org/) called “Protected Research Information Management Environment (PRIME)”. The PRIME instance is written in PHP, based on an objected-oriented programming (OOP) architecture, and JavaScript with inverted file indexing search algorithms to render the graphical user interface (GUI) (http://www.php.net/). PRIME has a user-friendly interface that is menu driven with help features that assist the user in navigating through the application. PRIME exploits the MySQL database system and allows powerful SQL queries across tasks through using fully normalized table structures (http://www.mysql.com/). It is a web-enabled application that runs on most popular browsers. It is provided to the clinical and translational science community as a free and easily customized database solution.
